# Effect of multiple intravitreal injections of conbercept on the cornea in patients with branch retinal vein occlusion-induced macular edema

**DOI:** 10.3389/fmed.2025.1595543

**Published:** 2025-06-25

**Authors:** Gaixia Zhai, Tiehui Shen, Yuanzhen Su, Na Liu

**Affiliations:** Zibo Central Hospital, Zibo, China

**Keywords:** conbercept, branch retinal vein occlusion, macular edema, cornea, best-corrected vision acuity

## Abstract

**Background:**

To investigate the effect of multiple intravitreal injections of conbercept on the cornea in patients with branch retinal vein occlusion (BRVO)-induced macular edema.

**Methods:**

The retrospective study analyzed the clinical data of 40 patients (40 eyes) with BRVO-induced macular edema between March 2020 and March 2023. All patients received intravitreal injections of conbercept according to the “3 + PRN” regimen and were followed for at least 6 months. Corneal analysis was performed using a corneal endothelial microscope and a Pentacam corneal topographic map. The best-corrected vision acuity (BCVA), central retinal thickness, central corneal thickness, corneal endothelial cell density, hexagonal cell percentage, and coefficient of variation before and at 1, 3, and 6 months after the injection were compared.

**Results:**

The study included 40 patients (22 males and 18 females) with an average age of 50.80 ± 7.35 years (range, 38–67 years). The corneal endothelial cell densities, central corneal thicknesses, hexagonal cell percentages, and coefficients of variation before and at 1, 3, and 6 months after the injection were not significantly different (*P* > 0.05). The average BCVA was significantly higher at 1, 3, and 6 months after than before the injection (*P* < 0.05 each). The average central retinal thickness was significantly lower at 1, 3, and 6 months after than before the injection (*P* < 0.05 each). The number of injections was 3.08 ± 0.27 at the last follow-up. No adverse reactions, such as endophthalmitis, retinal detachment, or thrombus, were observed in any patient after treatment.

**Conclusion:**

Multiple intravitreal injections of conbercept can improve the BCVA and reduce macular edema in patients with BRVO and have no significant effect on the cornea.

## 1 Background

Retinal vein occlusion (RVO) is the second most common retinal vascular disease after diabetic retinopathy. It is clinically characterized by tortuous dilatation of retinal veins and edema, bleeding, and exudation in the areas distributed along the veins (1). Depending on the location of the occlusion, RVO can be divided into branch (BRVO) and central. Arteriovenous cross-compression is the predominant pathogenic mechanism underlying BRVO. The pathogenesis of RVO is still unclear, and the main risk factors include hypertension, hyperlipidemia, diabetes, and systemic inflammation. Neovascularization and macular edema (ME) are the main complications of RVO, but ME is the main cause of vision loss (2–5). Persistent ME and the resulting damage to the fundal structure may lead to irreversible impairment of visual function.

Vascular endothelial growth factor (VEGF) plays an important role in the pathogenesis of ME secondary to BRVO (6). The main treatment for BRVO-induced ME has been anti-VEGF therapy (7–9). In clinical practice, the commonly used anti-VEGF drugs include ranibizumab (Lucentis; Genentech Inc., South San Francisco, CA, United States), conbercept (KH902; Chengdu Kanghong Biotech Co., Ltd., Sichuan, China), aflibercept (EYLEA; Bayer HealthCare, Berlin, Germany), and bevacizumab (Avastin; Genentech Inc.). Conbercept is a fusion protein composed of extracellular domain 2 of VEGF receptor (VEGFR)-1 and extracellular domains 3 and 4 of VEGFR-2, which are fused with the Fc fragment of human immunoglobulin G1 (IgG1).

Clinical studies have reported that VEGF and anti-VEGF drugs may affect the cornea, in addition to neovascular diseases, which should arouse the attention of clinicians (10). It has been reported that corneal endothelial cell density (ECD) in patients with age-related macular degeneration can be significantly reduced by ranibizumab and aflibercept (11). However, other studies have found that ranibizumab and bevacizumab are not toxic to corneal endothelial cells (12). At present, there are few reports about the effects of intravitreal injections of conbercept on the cornea.

This study aimed to ascertain the effect of multiple intravitreal injections of conbercept on the cornea in patients with BRVO-induced ME.

## 2 Materials and methods

### 2.1 Study protocol

The study received ethical approval from an institutional review board (IRB). All patients provided informed consent.

### 2.2 Patients

The clinical data of 40 patients (40 eyes) with BRVO-induced ME admitted to the Department of Ophthalmology of Zibo Central Hospital between March 2020 and March 2023 were retrospectively analyzed. All patients received intravitreal injections of conbercept according to the 3 + pro re nata (PRN) treatment strategy and were followed for at least 6 months.

The inclusion criteria were as follows: (a) patients who were clinically diagnosed with BRVO and needed anti-VEGF therapy; (b) patients who had not received any intraocular injection before; (c) patients with no history of wearing contact lenses; (d) no history of eye diseases affecting corneal endothelial function; (e) no history of eye surgery such as those for the internal eye and ocular surface; and (f) no history of ocular laser therapy within 3 months before anti-VEGF injection. The exclusion criteria were: (a) patients with any corneal or ocular surface disease; (b) patients with concurrent retinal or optic nerve diseases; (c) endothelial cell count less than 1000/mm^2^; (d) patients with incomplete clinical data; (e) age of < 18 years; (f) patients who had received anti-VEGF drugs, triamcinolone, or laser photocoagulation therapy.

### 2.3 Examination and treatment

The examinations included tonometry, best-corrected visual acuity (BCVA) (logMAR), slit lamp biomicroscopy, corneal endothelial microscopy, ocular fundus examination, and optical coherence tomography at baseline and 1, 3, and 6 months. Intraocular pressure was measured using non-contact tonometry (TX-20, Canon, Japan). A corneal endothelial microscope (KONAN MEDICAL, INC.) was used to determine the ECD, proportion of hexagonal cells (Hex%), and coefficient of variation (CV). Central corneal thickness (CCT) was measured with a Pentacam corneal topographic map (OCULUS Optikgerate GmbH). The inspection area was the central cornea, and the clearest image quality was taken to record the data. Central retinal thickness (CRT) was measured using optical coherence tomography (Optovue, Inc., Fremont, CA, United States). The device had an accompanying program to measure the CRT (distance from the inner boundary membrane of the retina to the retinal pigment epithelium), which is the thickness of the retinal nerve epithelium.

All patients received levofloxacin eye drops four times a day for 7 days before the injection, and all intravitreal injections were administered in the operating room of the bacteria-free laminar Flow center. The patient was placed in a supine position and administered surface anesthesia. The conjunctival sac was rinsed with Povidone iodine solution for 3 min, followed by 100 mL of normal saline. A 30G sterile syringe needle was inserted into the vitreous at 3.5–4.0 mm behind the corneal limbus perpendicular to the sclera, and conbercept (0.05 mL containing conbercept 0.5 mg) was injected into the vitreous body. A sterile cotton swab was used to press the tip of the needle subsequently, and tobramycin and dexamethasone eye ointments were applied and covered with gauze. All injection procedures were performed by the same ophthalmologist to minimize procedural variability. The tobramycin and dexamethasone eye ointments were also applied to the conjunctival sac, and gauze was used to cover the operative eye for 1 day. Levofloxacin eye drops were administered for 1 week after the procedure. The first follow-up was scheduled a week after the injection, and the follow-up frequency was once a month.

The criteria for reinjection included an increase in CRT by > 100 μm, new retinal fluid, a decrease in BCVA by more than one row, or new areas of retinal neovascularization.

### 2.4 Observed parameters

The BCVA (logMAR), CRT, CCT, ECD, Hex%, and CV were compared before and at 1, 3, and 6 months after the injection.

### 2.5 Statistical analysis

SPSS17.0 statistical software was used for the analysis. Quantitative data are expressed as mean ± standard deviation (SD). The BCVA was converted to the logarithm of the minimum angle of resolution (logMAR). Repeated-measures analysis of variance (ANOVA) was used to compare the changes in BCVA, CRT, CCT, ECD, Hex%, and CV before and at 1, 3, and 6 months after the injection. If the results of ANOVA were significant, the LSD-*t*-test was used for pairwise comparison of the sample means. *P*-values of < 0.05 denoted statistical significance.

## 3 Results

### 3.1 Baseline characteristics

The study included 40 patients (22 males and 18 females) with an average age of 50.80 ± 7.35 years (range, 38–67 years).

### 3.2 BCVA

The baseline BCVA was 0.55 ± 0.13 logMAR, decreasing significantly to 0.34 ± 0.13, 0.31 ± 0.13, and 0.27 ± 0.11 logMAR at 1, 3, and 6 months, respectively (*P* < 0.05) (Table 1).

**TABLE 1:** Best-corrected visual acuity (BCVAs), CRTs, CCTs, ECDs, Hex% values, and CVs before and after the injection.

Time	BCVA (LogMAR)	CRT (μm)	CCT (μm)	ECD (cells/mm^2^)	HEX (%)	CV (%)
Preoperative	0.55 ± 0.13	527.7 ± 33.63	529.5 ± 27.32	2565 ± 323.7	52.03 ± 6.14	37.23 ± 5.63
1 month	0.34 ± 0.13*	330.9 ± 35.74*	528.9 ± 27.02	2562 ± 319.2	51.58 ± 6.08	36.75 ± 5.47
3 months	0.31 ± 0.13*	269.7 ± 50.18*	528.4 ± 26.52	2560 ± 323.1	51.53 ± 5.74	36.73 ± 5.40
6 months	0.27 ± 0.11*	270.0 ± 62.44*	528.7 ± 26.33	2560 ± 322.3	51.63 ± 6.02	36.80 ± 5.68
*P*-value	0.000	0.000	0.072	0.135	0.174	0.068
*P*-value (1 month vs. 3 months)	0.135	0.000	–	–	–	–
*P*-value (1 month vs. 6 months)	0.004	0.000	–	–	–	–
*P*-value (3 months vs. 6 months)	0.056	0.979	–	–	–	–

BCVA, best-corrected visual acuity; logMAR, logarithm of the minimal angle of resolution; CRT, central retinal thickness; CCT, central corneal thickness; ECD, endothelial cell density; HEX (%), hexagonal cell percentage; CV, coefficient of variation; *P = 0.000 VS. preoperative.

### 3.3 CRT

The average CRT was 527.7 ± 33.63 μm before treatment, decreasing sig nificantly to 330.9 ± 35.74 μm, 269.7 ± 50.18 μm, and 270.0 ± 62.44 μm at 1, 3, and 6 months, respectively (*P* < 0.05) (Table 1).

### 3.4 CCT, ECD, Hex%, and CV

The average CCT of the affected eye was 529.5 ± 27.32 μm before the injection and 528.9 ± 27.02 μm, 528.4 ± 26.52 μm, and 528.7 ± 26.33 μm, at 1, 3, and 6 months after the injection, respectively. The CCT did not change significantly at any of the time points (*P* > 0.05). The average ECD of the affected eye was 2565 ± 323.7 cells/mm^2^ before the injection and 2562 ± 319.2 cells/mm^2^, 2560 ± 323.1 cells/mm^2^, and 2560 ± 322.3 cells/mm^2^ at 1, 3, and 6 months after the injection, respectively. The ECD did not change significantly at any of the time points (*P* > 0.05). The average Hex% of the affected eye was 52.03 ± 6.14 before the injection and 51.58 ± 6.08, 51.53 ± 5.74, and 51.63 ± 6.02, at 1, 3, and 6 months after the injection, respectively; the Hex% did not change significantly at any of the time points (*P* > 0.05). The average CV of the affected eye was 37.23 ± 5.63 before the injection and 36.75 ± 5.47, 36.73 ± 5.40, and 36.80 ± 5.68 1, 3, and 6 months after the injection, respectively; it did not change significantly at any time point (*P* > 0.05) (Table 1).

### 3.5 Complications

Over the six postoperative months, subconjunctival hemorrhage was observed in four patients, transient intraocular pressure elevation occurred in one patient, and corneal epithelial injury was observed in one patient. None of the patients included in this study had severe ocular or systemic adverse reactions related to the intravitreal injection or drugs.

### 3.6 Number of injections

Over the six postoperative months, the average number of injections was 3.08 ± 0.27.

## 4 Discussion

Branch retinal vein occlusion has various ocular sequelae, with ME being the most common. In patients with BRVO, retinal photoreceptor function is impaired due to the dysfunction of macular cells, which can further cause visual loss if not controlled in time. Therefore, effectively reducing the degree of retinal ischemia and hypoxia and accelerating the absorption of macular edema have become the key principles of the clinical treatment of BRVO-induced ME. It has been suggested that exudate accumulation (extracellular edema) caused by blood-retinal barrier (BRB) dysfunction is the main pathological mechanism underlying macular edema (13). VEGF may destroy the BRB and cause macular edema (14). For a long time, macular grating photocoagulation has been mainly used to treat ME secondary to BRVO. Despite its efficacy, it may damage visual cells, and it has limited effect on improving the visual acuity of patients (15). It has been gradually replaced by intravitreal injection of anti-VEGF drugs (16–19) and anti-inflammatory therapy (20, 21).

Intravitreal injection of anti-VEGF drugs not only effectively reduces macular edema, but also improves the visual acuity of patients (22). The principle is that the drug can reduce the VEGF concentration in the eye, inhibit the formation of new blood vessels, and reduce the permeability of blood vessels, thereby reducing the leakage of blood vessels and promoting the absorption of edema.

Corneal endothelial cells play an important role in maintaining corneal transparency, and corneal transparency is one of the important conditions for the normal physiological function of visual organs. As the innermost layer of the cornea, the corneal endothelium is composed of a single layer of endothelial cells, which are in contact with the anterior aqueous humor. Some scholars have found that corneal endothelial cells can express VEGF receptors (23). Some scholars have reported that the presence of anti-VEGF drugs can be detected in the anterior chamber water after an intravitreal injection (24). Therefore, the effect of multiple intravitreal injections of anti-VEGF drugs on the cornea requires further study.

The corneal morphology and CCT were not significantly affected by the short-term injection of aflibercept in a study (25). However, other scholars have come to a different conclusion (26).

Conbercept is a recombinant fusion protein produced using the hamster ovarian cell system. It exhibits a high affinity for VEGF and tightly binds to various VEGF-A subtypes, as well as VEGF-B and PIGF simultaneously (27). Conbercept is currently widely used to treat diabetic ME (DME) and ME secondary to RVO. A large number of studies have shown that conbercept is effective in treating RVO (28–31). However, we should be wary of the effect of conbercept on the cornea in the clinic.

To our knowledge, this study is the first to ascertain the safety of conbercept in the treatment of patients with ME secondary to BRVO using corneal endothelial microscopy and a Pentacam corneal topographic map.

In this study, 40 patients with BRVO-induced ME received intravitreal injections of conbercept according to the “3 + PRN” regimen and were followed for at least 6 months. The BCVA, CRT, CCT, ECD, Hex%, and CV were compared before and 1, 3, and 6 months after the injection. The results of this study showed that the ECD, Hex%, and CV at 1, 3, and 6 months after were not statistically significantly different from those before the injection. This suggests that conbercept has no significant effect on the corneal endothelium in patients with BRVO. There were no significant differences between the CCTs before and after treatment. This is not entirely consistent with the results of previous studies on the effects of ranibizumab and aflibercept on the cornea of patients with age-related macular degeneration (11). However, the conclusions of this study are consistent with previous studies on the effect of ranibizumab on the corneal endothelium in patients with age-related macular degeneration (32). In addition, the results of this study also showed that BCVA and CRT at 1, 3, and 6 months after treatment were significantly different from those before treatment. This suggests that conbercept can reduce ME in patients with BRVO and improve the BCVA. This is consistent with the reports of previous studies (33, 34). None of the patients included in this study had severe ocular or systemic adverse reactions related to intravitreal injection or drugs, indicating the safety of this treatment.

The main limitations of this study include its retrospective nature, small sample size, and short follow-up duration. While the PRN (pro re nata) regimen in the study reflects real-world clinical practice, it may introduce variability in treatment intervals. The absence of a fixed-dosing control group limits direct comparisons of treatment efficacy and safety. This retrospective study relied on existing data, which may be subject to incompleteness or missing records. The small sample size increases susceptibility to random variations, potentially compromising the stability and reliability of the conclusions. Additionally, the short follow-up duration might be insufficient to fully evaluate the long-term effects of the treatment. The next step is to increase the sample size, extend the follow-up duration, and conduct prospective multi-center randomized controlled trials to validate the findings.

## 5 Conclusion

Multiple intravitreal injections of conbercept can improve the BCVA and reduce macular edema in patients with BRVO. They demonstrated no significant effect on the morphology of corneal endothelium and CCT.

## Data Availability

The raw data supporting the conclusions of this article will be made available by the authors, without undue reservation.
